# Co-targeting aurora kinase with PD-L1 and PI3K abrogates immune checkpoint mediated proliferation in peripheral T-cell lymphoma: a novel therapeutic strategy

**DOI:** 10.18632/oncotarget.22222

**Published:** 2017-11-01

**Authors:** Shariful Islam, Eric Vick, Bryan Huber, Carla Morales, Catherine Spier, Laurence Cooke, Eric Weterings, Daruka Mahadevan

**Affiliations:** ^1^ Cancer Biology GIDP, University of Arizona Cancer Center, Tucson, AZ 85724, USA; ^2^ West Cancer Center and University of Tennessee Health Sciences Center, Memphis, TN 38163, USA; ^3^ Department of Hematopathology, University of Arizona, Tucson, AZ 85724, USA; ^4^ Department of Medicine, The University of Arizona Cancer Center, Tucson, AZ 85724, USA; ^5^ Department of Radiation Oncology, University of Arizona, Tucson, AZ 85724, USA

**Keywords:** T-cell lymphoma, immune checkpoint, aurora kinases, anti-PD-L1, PI3K isoforms

## Abstract

Peripheral T-cell non-Hodgkin lymphoma (PTCL) are heterogeneous, rare, and aggressive diseases mostly incurable with current cell cycle therapies. Aurora kinases (AKs) are key regulators of mitosis that drive PTCL proliferation. Alisertib (AK inhibitor) has a response rate ∼30% in relapsed and refractory PTCL (SWOG1108). Since PTCL are derived from CD4^+^/CD8^+^ cells, we hypothesized that Program Death Ligand-1 (PD-L1) expression is essential for uncontrolled proliferation. Combination of alisertib with PI3Kα (MLN1117) or pan-PI3K inhibition (PF-04691502) or vincristine (VCR) was highly synergistic in PTCL cells. Expression of PD-L1 relative to PD-1 is high in PTCL biopsies (∼9-fold higher) and cell lines. Combination of alisertib with pan-PI3K inhibition or VCR significantly reduced PD-L1, NF-κB expression and inhibited phosphorylation of AKT, ERK1/2 and AK with enhanced apoptosis. In a SCID PTCL xenograft mouse model, alisertib displayed high synergism with MLN1117. In a syngeneic PTCL mouse xenograft model alisertib demonstrated tumor growth inhibition (TGI) ∼30%, whilst anti-PD-L1 therapy alone was ineffective. Alisertib + anti-PD-L1 resulted in TGI >90% indicative of a synthetic lethal interaction. PF-04691502 + alisertib + anti-PD-L1 + VCR resulted in TGI 100%. Overall, mice tolerated the treatments well. Co-targeting AK, PI3K and PD-L1 is a rational and novel therapeutic strategy for PTCL.

## INTRODUCTION

Peripheral T-cell non-Hodgkin lymphomas (PTCL) belong to a rare (∼15% of NHL) and heterogeneous group of lymphoid malignancies, comprised of several subtypes: extra-nodal (NK/T-cell, nasal-type, enteropathy-type, hepatosplenic, subcutaneous panniculitis-like) and nodal PTCL (NOS) (not otherwise specified), anaplastic large cell lymphoma (ALCL, ALK-negative) and angioimmunoblastic T-cell lymphoma (AITL) [1, 2]. PTCL’s have a poor prognosis due to a very aggressive disease course and they are mostly incurable with current anti-proliferative therapies [3]. Aurora kinases (AKs) are key molecular and cellular drivers of PTCL proliferation [4]. AKs are mitotic serine/threonine kinases and 3 homologous isoforms (A, B/C) have been identified. AK-A localizes to centrosomes and the proximal mitotic spindle during mitosis. This enzyme is critical to bipolar spindle formation but also participates in centrosome maturation, separation, as well as mitotic entry. AK-B/C are chromosomal passenger proteins critical to chromosome alignment and cytokinesis [5]. Over-expression of AK-B > A is observed in PTCL cell lines and in human PTCL samples [4, 6]. AKs are validated therapeutic targets in pre-clinical studies [4, 7] and in clinical trials [6, 8]. An AK-A inhibitor, alisertib, reversed the deleterious effects of AKs on genetic instability, proliferation and anti-apoptosis. Although protein levels of AK-A and/or B do not appear to predict for an alisertib response, there is a ∼30% response rate to alisertib as a single-agent in relapsed and refractory PTCL [6].

PTCL’s are a heterogeneous collection of malignancies originating from CD4 and CD8 T-cells, however the role of immune checkpoints in the pathogenesis of PTCL is not well established. Targeting PD-1 (Program Death-1) in Hodgkin lymphoma and follicular lymphoma have shown sustainable responses [9], however little information is available for PTCL. B- and T-NHL display a fundamentally different biology with respect to PD-1 and PD-L1/L2 (Program Death Ligand -1/2) functions. In addition, the tumor microenvironment (TME) plays an important role in PTCL with tumor infiltrating T-cells (TILs) and other immune cells contributing to a pro-tumor immune suppression. It has been established that multiple receptor-mediated signaling pathways, including PI3K/mTOR, induce PD-L1 expression [10]. PI3-like kinases (PI3K’s) are central signaling mediators in NHL which activate cell proliferation and modulate immune regulation within the TME. AK inhibition activates PI3K through the mTOR pathway [11] and increases PD-L1 expression in PTCL.

We *hypothesize* that immune checkpoint mediated PTCL proliferation is amenable to targeting by inhibiting AK and PI3K and that these targets can be developed as a novel therapeutic strategy.Immunohistochemistry (IHC) of tumor samples from patients treated with alisertib for relapsed and refractory PTCL (SWOG 1108) [6] showed high Ki-67 and a PD-L1:PD-1 staining ratio of 8.9 fold. Here we report that PTCL cell lines showed high PD-L1 expression relative to PD-1 expression by Western blotting. *In vitro*, alisertib plus PI3Kα isoform and pan-inhibitors showed synergistic anti-PTCL activity in CRL-2396 cells that were synthetic lethal with vincristine (VCR). Syngeneic immune competent PTCL mouse xenograft models showed synthetic lethality of alisertib plus anti-PD-L1 therapy. This effect was further enhanced by pan-PI3K inhibition ± VCR in this mouse xenograft model. Together, our data suggests that targeting immune escape associated proliferation is a key concept to effective PTCL therapy.

## RESULTS

### PD-L1 is differentially expressed relative to PD-1 in PTCL

**Figure 1:**
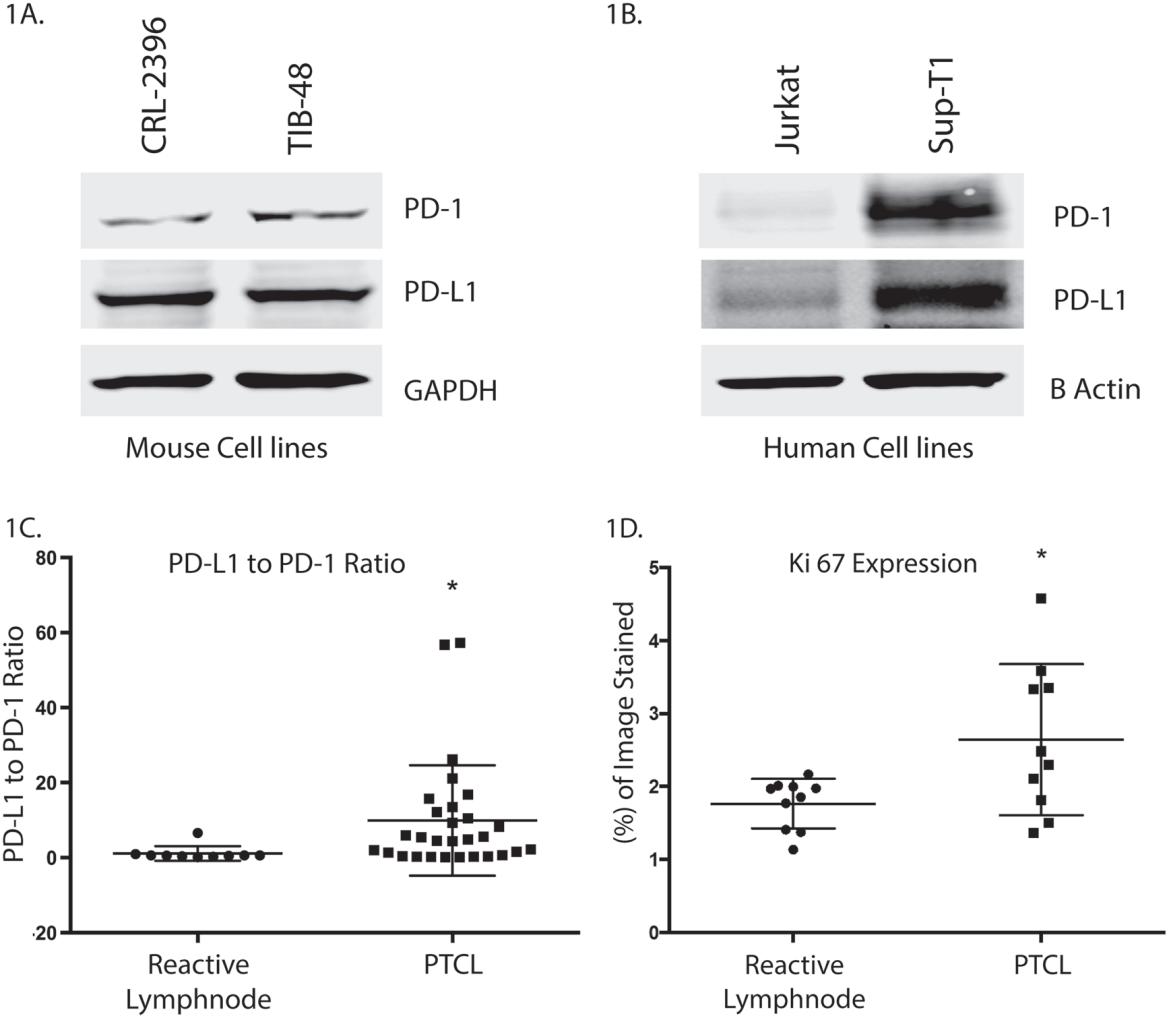
PD-L1 is differentially over-expressed relative to PD-1 in PTCL Mouse **(A)** and human **(B)** PTCL cell lines over-express PD-L1 relative to PD-1 by Western blotting. CRL-2396, TIB-48, Jurkat and SUP-T1 cells were lysed and probed by Western blotting PD-L1 and PD-1. The ratio of PD-L1: PD-1 is high. IHC analysis of PD-L1 and PD-1 from SWOG1108 shows reactive lymph node control has a low PD-L1: PD-1 ratio. In contrast, PTCL samples show a ∼9-fold increased ratio for PD-L1: PD-1 expression **(C)** and ki-67 over-expression **(D)**.

Targeting PD-1 (Program Death-1) in Hodgkin lymphoma and follicular lymphoma have shown sustainable responses however the role of immune checkpoints in the pathogenesis of PTCL is not well established. Western blotting demonstrated that the PTCL cell lines CRL-2396, TIB-48 and SUP-T1 over-express PD-L1 relative to PD-1 (Figure 1A, 1B). Similarly, IHC of pre-alisertib PTCL patient biopsies (n=22) from the SWOG1108 study reveals that PD-L1 is also over-expressed relative to PD-1 with a staining ratio of 8.9 fold (p=0.0037) in favor of PD-L1 (Figure 1C) and Ki-67 (Figure 1D) compared to a normal lymph node control is an indication of aggressiveness of this disease.

### Pan-PI3K inhibition ± VCR is synergistic with AK inhibition in PTCL cell lines

**Figure 2:**
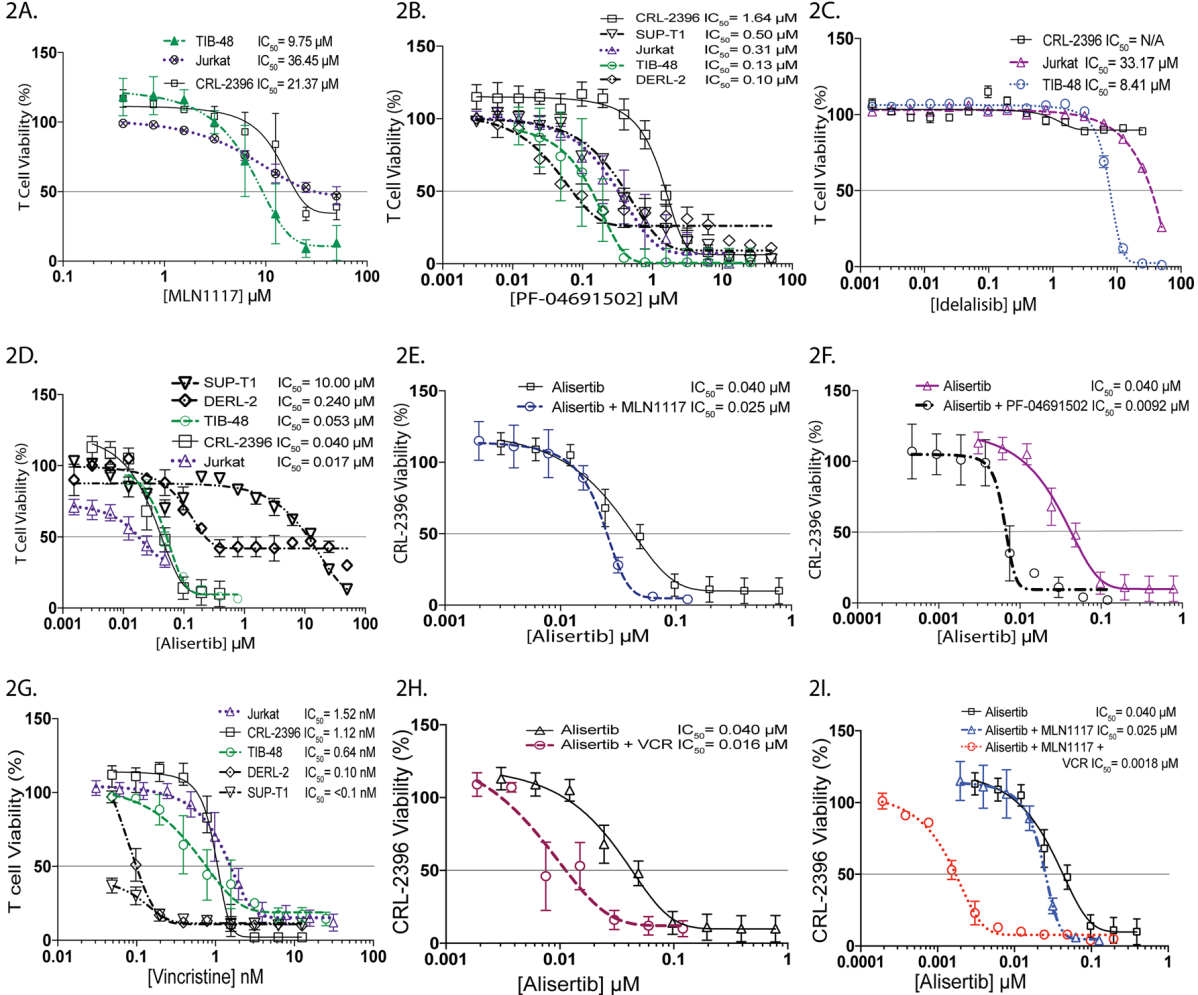
Pan-PI3K inhibition ± VCR is synergistic with AK inhibition in PTCL cell lines MTS cell viability assay of the PTCL cell lines were performed respectively for MLN1117 **(A)**, PF-04691502 **(B)**, Idelalisib **(C)**, Alisertib **(D)**, and Vincristine **(G)**. The combination of alisertib plus MLN1117 **(E)**, alisertib plus PF-04691502 **(F)**, alisertib plus Vincristine **(H)** and alisertib plus MLN1117 plus vincristine **(I)** in the CRL-2396 cell-line demonstrated synergistic interaction.

In relapsed and refractory PTCL, inhibition of PI3Kδ with CAL-101 (idelalisib) or IPI-145 has shown durable responses [12], most likely due to targeting of immune suppressive T-cells (T_REG_) in the Tumor Microenvironment (TME). Constitutively active PI3K plays key roles in G2/M transition and leads to defects in the DNA damage checkpoint control [13]. These observations prompted us to perform cell viability assays with single agent PI3K inhibitors targeting PI3Kα, PI3Kγδ and pan-inhibitor therapy in PTCL cell lines. The IC_50_ values for MLN1117 (a PI3Kα inhibitor) indicate a micro-molar differential activity: TIB-48 = 9.75μM, Jurkat = 36.45μM and CRL-2396 = 21.37μM respectively (Figure 2A). In contrast, PF-04691502 (a pan-PI3K inhibitor) yielded significantly lower IC_50s_ values: CRL-2396 = 1.64μM, SUP-T1 = 500nm, Jurkat = 310nM, TIB-48 = 130nM and DERL-2 = 100nM respectively (Figure 2B), indicating a 19-116 fold potency increase over PI3Kα inhibition. The PI3Kδ inhibitor idelalisib displayed no activity in CRL-2396 but had an IC_50_ value of 33.17μM in Jurkat cells and 8.41μM in TIB-48 cells (Figure 2C), similar to PI3Kα inhibition, which is consistent with a prior report [14].

Alisertib is active in all PTCL cell lines with an IC_50_ ranging from 17nM – 10.0μM (Figure 2D). Combination index (CI) (Table 1) using Chou-Talalay analysis confirmed additivity for alisertib + MLN1117 (PI3Kα inhibitor) (Figure 2E) but strong synergism for alisertib + PF-04691502 (pan-PI3K inhibitor) in CRL-2396 cells (Figure 2F). Vincristine (VCR) is an active agent in NHL particularly when used with combination chemotherapy (e.g. CHOP) or alisertib. In PTCL cell lines VCR had an IC_50_ of <0.1nM (SUP-T1), 0.10nM (DERL-2), 0.64nM (TIB-48), 1.12nM (CRL-2396) and 1.52nM (Jurkat) (Figure 2G). VCR plus alisertib is synergistic in CRL-2396 cells (Figure 2H). VCR plus PF-04691502 and VCR plus MLN1117 was also synergistic in Jurkat cells (Table 1). In addition, VCR plus alisertib plus MLN1117 showed high synergy shifting the cytotoxicity curve to the left by 100-fold in CRL-2396 cells (Figure 2I).

**Table 1:** Combination Index (CI) of targeted therapies for PTCL

Combination Index (CI) Values	CRL-2396	TIB-48	Jurkat
Alisertib + MLN1117	1.02	2.52	3.76
Alisertib + PF-04691502	0.46	1.62	1.86
Alisertib + Vincristine	0.79	3.17	0.72
MLN1117 + Vincristine	1.23	1.70	0.35
PF-04691502 + Vincristine	0.41	1.53	0.94
Alisertib + MLN1117 + Vincristine	0.10	0.63	0.80
Alisertib + PF-04691502 + Vincristine	N/A	2.30	0.48

NB. CI value <0.90 = Synergism; 0.90 - 1.10 = Additive; >1.10 = Antagonism

The combination index of different drug combinations (first column) are shown for CRL-2396 cells, TIB-48 cells and Jurkat cells respectively in 2nd, 3rd and 4th column which demonstrates synergy of interaction. (N/A: Not Available).

### A combination therapy of alisertib plus VCR plus PI3Kα inhibition displays strong anti-proliferative activity in a PTCL mouse xenograft model

**Figure 3:**
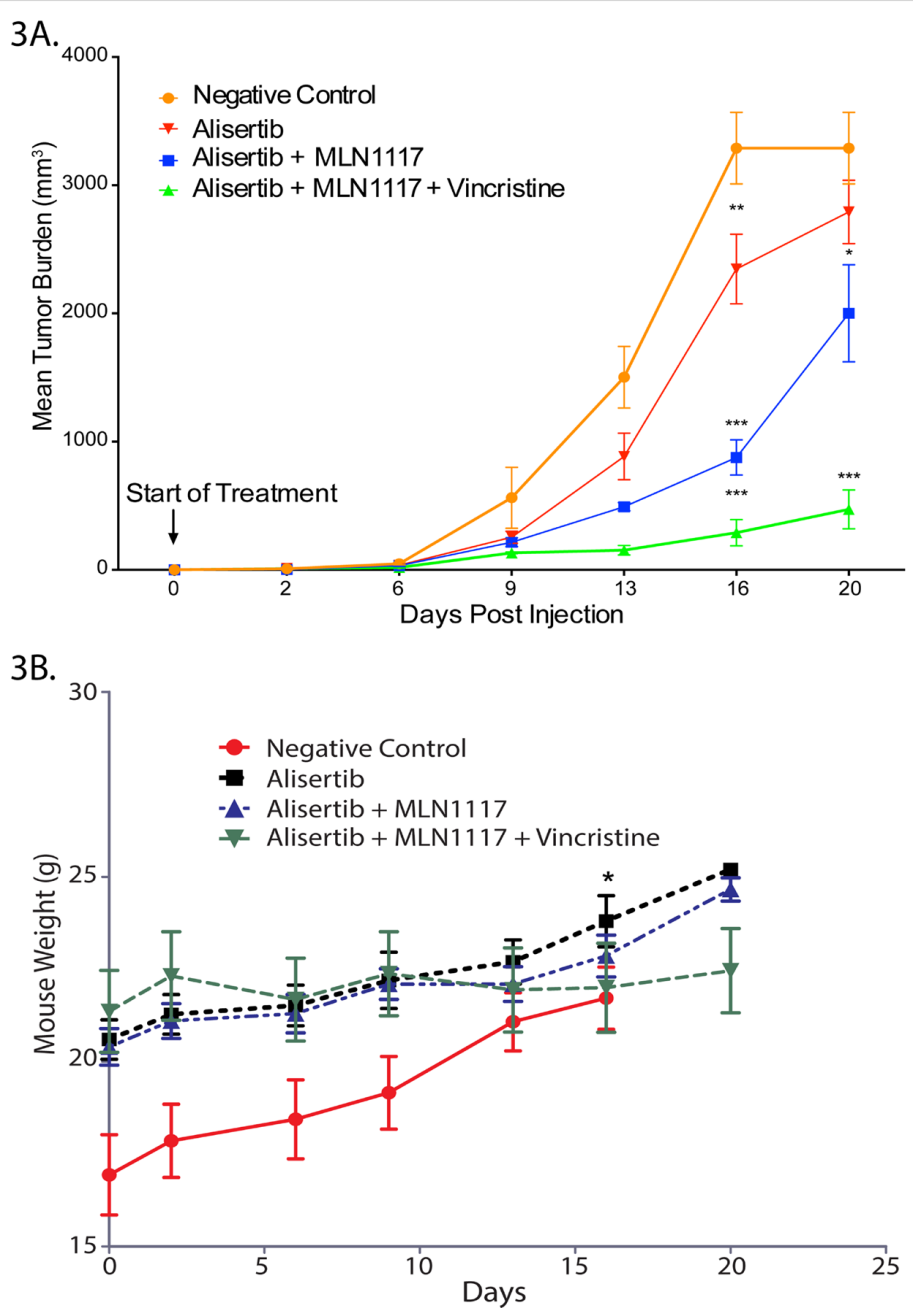
A combination therapy of alisertib plus VCR plus PI3Kα inhibition displays strong anti-proliferative activity in a PTCL mouse xenograft model A PTCL mouse xenograft model (CRL-2396 cells in BALB/c) evaluating AK + PI3Kα inhibition. There was a significant decrease in mean tumor burden between control versus alisertib alone on day 16 (mean decrease of 497mm^3^, p=0.012), versus the combination of alisertib + MLN1117 (mean decrease of 1288mm^3^ on day 16, p<0.001) on days 13, 16 and 20 **(A)**. All treatments were well tolerated with no body weight changes except on day-16 there was only significant changes between negative control group and alisertib as a single agent group with p=0.043 **(B)**.

Our, *in vitro* synergy studies prompted a mouse PTCL xenograft study to evaluate the above-described therapies for anti-tumor activity, tolerability and safety *in vivo*. The mouse PTCL CRL-2396 BALB/c xenograft model treated with alisertib (30 mg/kg daily oral x 3 weeks) ± PI3Kα inhibitor MLN1117 (30 mg/kg daily oral x 3 weeks) yielded a tumor growth inhibition (TGI) of ∼30% with alisertib alone versus control (mean decrease 497 mm^3^, p=0.012). Treatment with alisertib + MLN1117 resulted in a TGI of ∼60% (mean decreases 1288 mm^3^ p<0.001). The addition of VCR (0.375 mg/kg IV Q1W x 3) to alisertib plus MLN1117 resulted in a TGI of > 90% with a p<0.001 (Figure 3A) indicating anti-tumor activity as predicted by *in vitro* cell culture models. Mice were divided randomly (pair-matched) into different groups based on their tumor volume but independent of body weight which results in low weight mice grouped in negative control group. All treatments were well tolerated with no body weight changes except on day-16 there was only significant changes between negative control group and alisertib as a single agent group with p=0.043 (Figure 3B). This PTCL mouse xenograft study confirms that alisertib synergizes with PI3Kα inhibition and that VCR displays synthetic lethality with the alisertib plus MLN1117 combination.

### A combination therapy of alisertib plus VCR plus pan-PI3K inhibition induces apoptotic cell death

**Figure 4:**
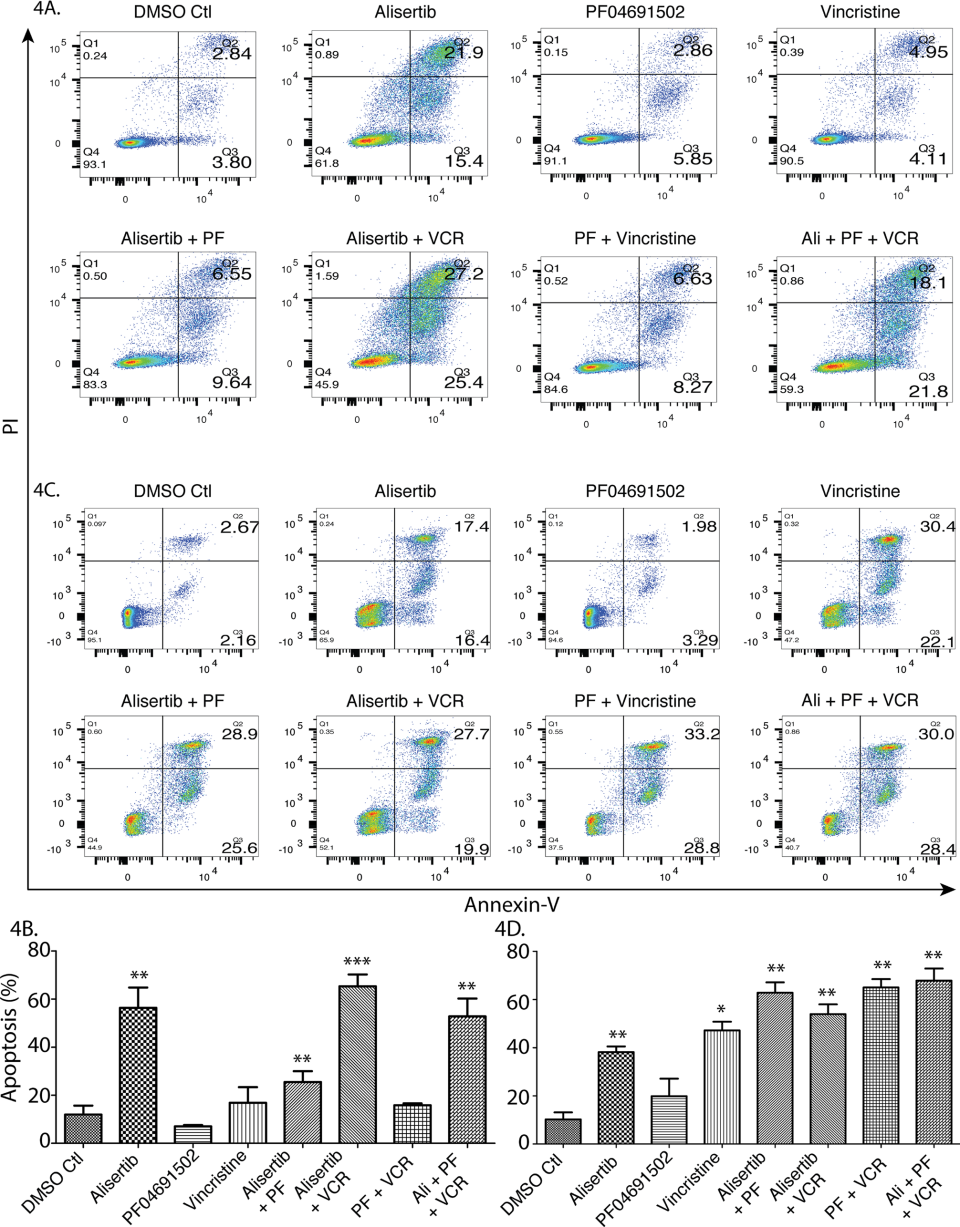
A combination therapy of alisertib plus VCR plus pan-PI3K inhibition induces apoptotic cell death CRL 2396 cells **(A)** were treated with 50nM alisertib, 1.5μM PF04691502, 0.75nM vincristine and their combination for 4 days and SUP-T1 cells **(C)** were treated with 50nM alisertib, 500nM PF04691502, 2.0nM vincristine and their combination for 4 days followed by Flow cytometry for analysis of apoptosis; quantified and expressed as mean ± S.E. in **(B)** and **(D)** respectively.

To investigate cell death mechanisms, the CRL-2396 (Figure 4A and 4B) and SUP-T1 (Figure 4C and 4D) cell lines were treated with single modality drugs and their combinations and evaluated for cellular necrosis versus apoptosis. We demonstrate that apoptosis is the cause of cell death. Though the pan-PI3K inhibitor (PF04691502) is very active as an anti-proliferative agent, it failed to induce apoptosis in *in vitro* cell culture assays (p=0.293 for CRL-2396, p=0.186 for SUP-T1), likely due to inhibition of cell growth. Vincristine synergizes with alisertib (p=0.0002 for CRL-2396, p=0.007 for SUP-T1) but triple therapy did not induce further synergism in CRL-2396 cells (p=0.004) and SUP-T1 cells (p=0.004) which may be due to growth inhibitory nature of PF04691502. However, this combination is more potent in *in vivo* mouse models (Figures 3 and 6).

**Figure 5:**
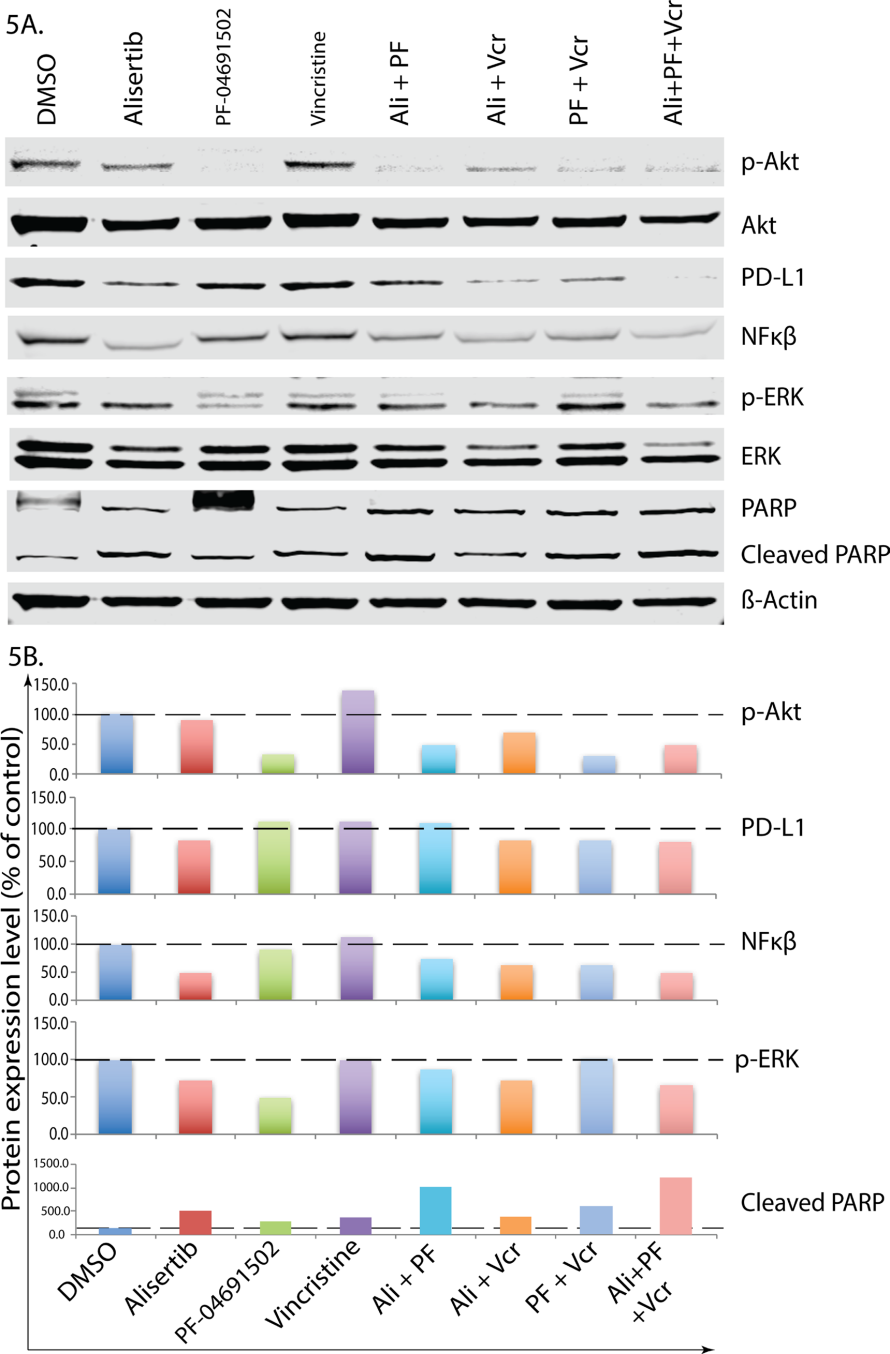
Alisertib plus VCR plus pan-PI3K inhibition abrogates PD-L1 induction and inhibits PTCL cell proliferation CRL 2396 cells were treated with 50nM alisertib, 1.5μM PF04691502, 0.75nM vincristine and their combination for 4 days followed by Western blotting analyses for protein expression **(A)** and quantification **(B)**.

**Figure 6:**
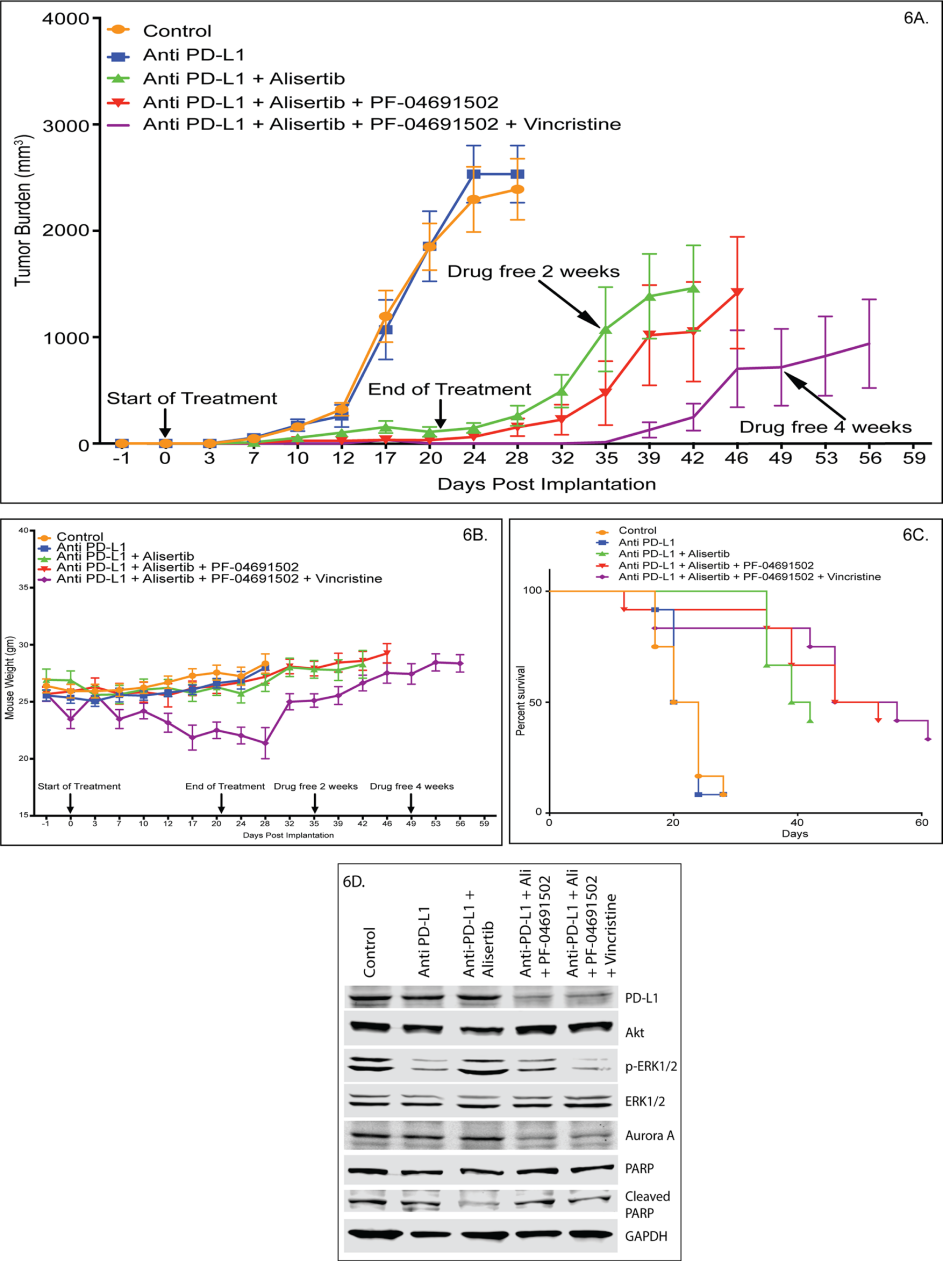
Combination of alisertib plus anti-PD-L1 Mab is highly synergistic and amplifies the anti-PTCL activity of pan-PI3K inhibition and VCR in a mouse xenograft model **(A)** ARK/J CRL-2396 syngeneic xenograft mice with flank tumors are treated with DMSO/buffer control versus anti-PD-L1 versus alisertib + anti-PD-L1 versus alisertib + anti-PD-L1 + PF-04691502 versus alisertib + anti-PD-L1 + PF-04691502 ± VCR. Tumor growth inhibition is shown with significant reduction on days 12-32 with alisertib + anti-PD-L1 (p<0.01), days 12-35 with alisertib + anti-PD-L1 + PF-04691502 (p<0.001) and days 7-56 on quadruple therapy (p<0.01). **(B)** Body weight changes on-treatment and post-treatment is shown for all arms with significant reduction at days 12, 17, 20 and 24 of quadruple therapy only (p<0.05). **(C)** Kaplan-Meier survival curves are shown for all arms where 4 drug combination showed statistically superior overall survival versus alisertib + anti-PD-L1 (p<0.0001). **(D)** Western blotting of harvested mouse tumors showed effectiveness of combination therapy.

### Combination therapy of Alisertib plus VCR plus pan-PI3K inhibition abrogates PD-L1 induction and inhibits PTCL cell proliferation

To further study the underlying mechanism of cell death signaling, CRL-2396 cells (Figure 5A) were treated with individual drugs, their combinations and analyzed for differential signaling protein modulation using Western blotting with quantification (Figure 5B). Alisertib plus VCR plus pan-PI3K inhibition significantly reduced the expression of PD-L1 and NF-κβ, which are key drivers PTCL proliferation. In addition, this combination also inhibits the phosphorylation AKT, ERK and enhances apoptosis evident by PARP cleavage. Adding anti-PD-L1 to the above 3 drug combinations *in vivo* further amplifies this process.

### Combination of alisertib plus anti-PD-L1 Mab is highly synergistic and amplifies the anti-PTCL activity of pan-PI3K inhibition and/or VCR in a mouse syngeneic xenograft model

Targeting immune suppression (PD-L1 and PI3Kγδ) and T-cell proliferation (AKs, PI3Kα) in PTCL is not well established. A pilot study showed that CRL-2396 cells when injected into the flank of BALB/c or ARK/J mice grew rapidly to >2500 mm^3^ within 1 week. Treatment with an anti-PD-L1 antibody (BE0101, BioXCell, NH) at different doses (50 μg and 100 μg IV Q1W x3) day 3 post-implantation of CRL-2396 cells were ineffective (data not shown). In a subsequent study in a ARK/J syngeneic mouse xenograft model, drug dosing was initiated at day 3 post-implantation and mice were pair-matched. Anti-PD-L1 Mab (BE0101, BioXCell, NH) given at 10 mg/kg, IP, every other day (QOD) x3 weeks was ineffective versus control (0.1% DMSO + saline) despite expression of PD-L1 on CRL-2396 cells. In contrast, alisertib (30 mg/kg daily oral, for 3 weeks) plus anti-PD-L1 Mab (10 mg/kg QOD IP, every other day x3 weeks) led to a TGI of >90%. However, 40% of mice relapsed within 2 weeks after discontinuation of therapy (Figure 6A). The addition of PF-04691502 (10mg/kg daily oral, x3 weeks) results in tumor regression. Once treatment ended 20% of mice relapsed within 2 weeks after ending treatment. At 4 weeks 50% of mice relapsed. In contrast, when VCR (0.375 mg/kg IV, Q1W x3) was added to the triple combination of alisertib plus anti-PD-L1 plus PF-04691502, tumor regression was observed and no relapsed occurred at 2 weeks after discontinuation of treatment. However, only 25% of mice relapsed at 4 weeks after discontinuation of treatment. All treatments were well tolerated with no changes in body weight except for the 4 drugs combination arm. However, after discontinuation of treatment these mice completely recovered their starting body weights (Figure 6B). Kaplan-Meier analysis of overall survival shows that mice treated with alisertib + anti-PD-L1 had a superior overall survival versus alisertib or anti-PD-L1 alone. In addition, alisertib + anti-PD-L1 + PF-04691502 ± VCR combinations showed statistically superior overall survival versus alisertib + anti-PD-L1 (p<0.0001) (Figure 6C). Western blotting of tumors harvested at the end of treatment showed that triple and quadruple combination therapies reduces PD-L1 and Aurora A expression (Figure 6D). These results indicate that alisertib alone has a response rate of ∼30%, while anti-PD-L1 alone was not active in PTCL. However, the alisertib + anti-PD-L1 combination is highly active and forms a backbone to target key components of immune escape (PI3K) and proliferation (VCR) in PTCL.

## DISCUSSION

The development of effective therapies for PTCL remain a serious challenge and the identification of novel targets to guide innovative treatment strategies should be a leading priority. The aurora kinase (AK) inhibitor alisertib is efficacious in rapidly proliferating tumors such as PTCL [4, 6, 8]. A ∼30% response rate of alisertib as a single agent was observed in a phase II [6] and phase III [15] trial of relapsed and refractory PTCL. We surmised that immune suppression is responsible to a large degree for the relative ineffectiveness of anti-proliferative therapies in PTCL. We sought to optimize the response to alisertib by investigating molecular and cellular determinants of immune suppression associated with PTCL proliferation driven by a pro-tumor cytokine milieu within the tumor microenvironment (TME). Here we address concurrent targeted therapeutic strategies directed toward immune suppression driven by PD-L1 modulation by the PI3K pathway, which is a key mechanism of resistance to existing therapies in PTCL progression.

IHC of patients with relapsed and refractory PTCL (SWOG 1108) [6] showed a high proliferative index with increased Ki-67 that correlates with increased AK expression [6]. In PTCL patients the PD-L1:PD-1 expression ratio was ∼9 fold (p=0.0037) in favor of high PD-L1 expression [16] indicating that malignant T-cells are innately equipped for immune suppression and escape from current therapies. All PTCL cell lines demonstrated increased PD-L1 expression relative to PD-1 expression, indicating an important role for PD-L1 in PTCL pathogenesis. Treatment of PTCL cells with alisertib showed continued PD-L1 expression, suggesting the requirement to inhibit immune suppression to enhance anti-PTCL activity with novel drugs. PD-L1 expression is induced by specific cytokines that activate the PI3K pathway allowing PTCL to evade the immune system by maintaining PD-L1 expression, a negative prognostic factor.

The PI3K pathway is known to induce PD-L1 expression [10] and to play key roles in the pathogenesis of lymphoma by regulating proliferation [17] and immune suppression within the TME. In chronic lymphocytic leukemia (CLL), diffuse large B-cell lymphoma (DLBCL) and mantle cell lymphoma (MCL), the PI3Kγδ isoforms are constitutively active and appear to be important in trafficking and infiltrating lymph nodes [18]. PI3Kγδ isoform inhibitors (e.g. idelalisib, duvelisib) block signals from the TME and enhance egress of malignant lymphocytes. Duvelisib a PI3Kγδ inhibitor with a 14-fold increased potency than idelalisib in inhibiting PI3Kγδ has shown activity in patients with T-cell lymphoma [12]. In the SWOG 1108 study of relapsed and refractory PTCL, we demonstrated that PI3Kδ is highly expressed in the cytoplasm of T-cells within the TME [6] but this did not correlate with an alisertib response. We investigated anti-PTCL activity of PI3K isoform (α, γδ) inhibitors and a pan-PI3K inhibitor alone and in combination with alisertib in PTCL cell lines. We demonstrate that the PI3Kγδ inhibitors had no anti-PTCL activity *in vitro* consistent with a prior report [14]. The PI3Kα inhibitor MLN1117 had modest anti-PTCL activity. In contrast, the pan-PI3K inhibitor PF-04691502 had superior activity. These results indicate that in PTCL cell lines, inhibiting all 3 PI3K isoforms are essential for anti-tumor activity. In addition, there is a strong synergism for alisertib + PF-04691502 versus alisertib + MLN1117 in CRL-2396 cells. However, addition of VCR optimally synergizes with AK plus PI3K inhibition.

Vincristine sensitizes AK inhibition with alisertib in PTCL. The shift in the cytotoxicity curve to the left observed in PTCL for alisertib plus VCR was also observed in MCL [7] and DLBCL [19] indicating the exquisite synergy is mechanistic of inhibiting mitotic proliferation by preventing spindle assembly during mitosis leading to mitotic catastrophe. Since alisertib is highly synergistic with PI3K inhibition as well as VCR, triple therapy demonstrated a further augmented synergy due to the enhanced effect on G1 and G2/M arrest leading to apoptosis. In DLBCL (*in vitro* and *in vivo*), AMG319 a PI3Kδ inhibitor plus VCR also demonstrated synergy with increased cytotoxicity [20]. Our mouse PTCL xenograft model showed that the PI3Kα inhibitor MLN1117 plus alisertib versus alisertib alone had superior TGI of 60% versus 30% respectively. However, the addition of VCR to alisertib plus MLN1117 showed a TGI of ∼90% confirming *in vitro* cell culture modeling for CRL-2396 cells.

The mechanistic link between immune suppression and proliferation in PTCL is not well established with respect to immune checkpoint regulators. Our syngeneic mouse PTCL xenograft model demonstrated that alisertib as a single agent had a TGI of ∼30% that significantly improved TGI to >90% when combined with an anti-PD-L1 monoclonal antibody which had no single agent activity. This exciting result indicates alisertib synergizes with anti-PD-L1 and supports the concept that immune escape by suppression is closely linked to mitotic proliferation that can be successfully co-targeted. However, 14 days after end of treatment 40% mice do relapse. An active PI3K pathway is known to induce the PD-1/PD-L1 axis promoting immune suppression [10]. Since alisertib also synergizes with PI3K inhibition, the addition of PF-04691502 a pan-PI3K inhibitor to alisertib + anti-PD-L1 antibody showed tumor regression but 14 days after discontinuing therapy 20% mice relapse. In contrast, VCR added to triple therapy lead to a deeper tumor regression with no mice relapsing at 2 weeks. The triple or quadruple therapy arms demonstrate mechanistic synergy due to co-targeting of immune escape/suppression simultaneously with proliferation. Hence, we surmised that pan-PI3K inhibition targets PTCL and suppressive T-cells (T_REG_) within the TME thereby altering the pro-inflammatory tumor promoting cytokine milieu. In CLL, lenalidomide (imid) causes a cytokine release syndrome due to up-regulation of PI3Kδ p110 with activation of AKT. However, Idelalisib plus lenalidomide dampens this immune response without compromising anti-tumor activity [21] providing a rationale for our observations.

In conclusion, we demonstrate that the AK inhibitor alisertib synergizes with anti-PD-L1 treatment thus providing a novel backbone therapy for PTCL. In addition, alisertib also synergizes with pan-PI3K inhibitor PF-04691502 targeting both PTCL and immune suppressive T-cells in the TME providing a second approach to PTCL therapy. Thirdly, alisertib has exquisite synergy with VCR further augmenting anti-PD-L1 and PI3K inhibition in PTCL. Since alisertib shifts the anti-proliferative curve to the left significantly with either VCR or pan-PI3K inhibition, the addition of anti-PD-L1 completes the blocking of the immune inhibitory loop enhancing the anti-tumor response in PTCL. We thus propose a novel therapeutic approach to treating PTCL with alisertib based synergistic combinations that target immune suppression (PD-L1, PI3Kγδ) and proliferation (PI3Kα inhibition, pan-PI3K inhibition and VCR) that could be evaluated in early phase therapeutic trials in PTCL patients relapsing after CHOP-like therapies.

## MATERIALS AND METHODS

### Cells and reagents

Peripheral T-cell non-Hodgkin lymphoma (PTCL) murine cell lines TIB-48 and CRL-2396 (TK1) and human leukemia cell line Jurkat clone E6-1 (ATCC) and SUP-T1, DERL-2 (DSMZ) were maintained in RPMI medium (Mediatech, VA) supplemented with 10% fetal bovine serum, 2mM sodium pyruvate at 37°C in a humidified atmosphere containing 5% CO_2_. Alisertib and MLN1117 were provided by Takeda pharmaceuticals (Cambridge, MA). PF-04691502, idelalisib, duvelisib and vincristine were purchased from SelleckChem (Houston, TX). Mouse Anti-PD-L1 (BE0101) antibodies were purchased from BioXCell (West Lebanon, NH).

### Immunohistochemistry

Paraffin-embedded sections were de-paraffinized and rehydrated with stepwise addition of distilled water. Antigen retrieval was carried out by bringing slides to a boil in 1 mM Sodium Citrate Buffer pH 6.0 followed by 20 min at a sub-boiling temperature. After washing in TBS three times, the slides were incubated in 3% hydrogen peroxide in TBS for 10 min and then blocked with blocking solution (TBS, 0.025% Triton-X100, 10% normal goat serum) for 2hr at room temperature. The slides were then immune-stained using 1:250 anti-PD-L1, 1:250 anti-PD-1, or 1:500 anti-Ki-67 antibodies in blocking solution. The reaction was incubated overnight at 4°C. After washing 3 times with TBS, the anti-rabbit or anti mouse HRP conjugated secondary antibodies (Santa Cruz Biotechnology, Dallas, TX) were incubated at a dilution of 1:500 in blocking solution for 2 hr at room temperature. The signal was checked using DAB staining (Sigma) following the manufacturer’s protocol. Primary or secondary antibody replacements with normal serum from the same animal species were used as negative controls. Analysis of samples was performed using IHC profiler on ImageJ to compare the staining density of DAB at 20x using spectral deconvolution with a pixel by pixel comparison [22]. Comparison ratio was calculated by comparing the mean staining intensity of each sample group.

### Cytotoxicity assays

PTCL cells were seeded at 8,000 per well in 96-well culture plates and allowed to grow for 24 hr followed by the desired treatment with increasing concentrations of the indicated agents for 4 days. Viable cell densities were determined using a CellTiter 96 Cell Proliferation Assay (Promega, Madison, WI). The studies were performed in 4 independent sets of triplicates repeat and IC_50_ values were estimated by Calcusyn software (Biosoft, UK). For combination studies of alisertib and PI3K inhibitor or vincristine, an equipotent ratio was calculated to determine a combined graded combination treatment. A control group was established for each drug treatment in six replicates. The effects of the combined treatments were determined by the combination-index (CI) and isobologram methods derived from the median-effect principle of Chou and Talalay [23].

### Apoptosis assays

Annexin V staining was used to detect apoptosis. Treated cells were harvested and rinsed with cold PBS once. After centrifugation for 5 min, cells were suspended in 500 μl of 1X Annexin V binding buffer (BioVision, Mountain View, CA, Annexin V-FITC Reagent Kit, Cat.#1001-1000) and then 5μl of Annexin V-FITC and 5μl of propidium iodide (BioVision, Annexin V-FITC Reagent Kit) were added. After incubation for 5 min at room temperature in the dark, the samples were analyzed by flow cytometry. All studies are performed in three or more biological replicates and then quantitatively expressed as mean±S.E.

### Immunoblotting

Cells are lysed in 1X RIPA buffer and supplemented 1:100 Protease/ Phosphatase inhibitor cocktail (Cell Signaling Technology, Danvers, MA). Protein concentrations are determined using the BioRad protein assay kit (Hercules, CA) and 30μg of protein resolved by electrophoresis on a 10% SDS-PAGE. The proteins are transferred onto a nitrocellulose membrane and nonspecific binding is blocked by incubating with 5% nonfat milk in TBS-T buffer (0.01 M Tris–Cl, 0.15 M NaCl, 0.5% Tween-20, pH 8.0) at room temperature for 1h. The membrane is subjected to the indicated antibodies and fluorescence detected using a LI-COR Odyssey Infrared Imaging System. Mouse Anti-PD-L1 (BE0101), mouse anti-PD-1(BE0146) and control rat IgG2A (BE0090) antibodies were purchased from BioXCell (West Lebanon, NH). Anti-Ki-67 (sc-15402) was obtained from Santa Cruz Biotechnology (Dallas, TX). Human Anti-PD-L1 (CST #13684), human anti-PD-1 (CST #86163), Anti-aurora A (CST #14475), Anti-phospho-aurora A (Thr288) (CST #3079), anti-Akt (CST #4691), anti-phospho-Akt (Ser473) (CST #4060), anti-ERK1/2 (CST #4695), anti-phospho-ERK1/2 (CST #4370), anti-PD-L1 (human) (CST #13684), anti-NFκβ (CST #8242) and anti-GAPDH (14C10) (CST #2118), anti-β-Actin (CST #3700) antibodies were purchased from Cell Signaling Technology (Danvers, MA).

### PTCL mouse xenograft model

Animal care and treatment were performed at Arizona Cancer Center’s experimental mouse shared services (EMSS) core facility. In the first study, BALB/c mice were injected with 1×10^6^ CRL-2396 cells subcutaneously into the right hind flank. In the second study AKR/Jmice were injected with 1×10^6^ CRL-2396 cells subcutaneously into the right hind flank. Three days after the initial injection, mice were divided randomly (pair-matched) into different groups with 12 mice per cohort. The first study evaluated control (PBS) versus alisertib (30 mg/kg PO QD x 3 weeks) versus alisertib plus MLN1117 (30 mg/kg PO QD x 3 weeks) versus alisertib plus MLN1117 plus vincristine (0.375 mg/kg IV once/week, ×3 weeks).

The second study treated mice with intraperitoneal (IP) control antibody (IgG with DMSO) versus anti-PD-L1 antibody alone (10mg/kg QOD IP, every other day x 3 weeks) versus anti-PD-L1 plus alisertib (30 mg/kg PO QD x 3 weeks) versus anti-PD-L1 plus alisertib plus PF-04691502 (10 mg/kg QD) versus anti-PD-L1 antibody plus alisertib plus PF-04691502 and vincristine (0.375 mg/kg IV once/week, × 3 weeks). The length (L) and width (W) of the subcutaneous tumors were measured by calipers and the tumor volume (T_V_) was calculated as: T_V_ = (L×W^2^)/2. Mice were sacrificed at the end of treatment (3 mice per cohort), end of study or if they reached >2000 mm^3^ at any time during the study.

### Statistical analysis

*In vitro* experiments were performed in triplicate. The data were expressed as mean ± S.E. and analyzed by student t-test unless otherwise indicated. Tumor growth was analyzed by two-way ANOVA with a test of multiple comparisons compared to the mean of the control to assess overall significance as well as student t-tests to assess individual p values. Survival of the mice was measured from the date of pair matching to sacrifice (event) or end of study (censored). The Kaplan-Meier method was used to estimate survival. The log rank test was used to compare survival between the respective treatment groups. Statistical adjustments were made for multiple comparisons. Analysis was performed using Prism (Graphpad, La Jolla, CA). All p-values ≤0.05 were considered statistically significant.
